# Mexican Hat Modulation of Visual Acuity Following an Exogenous Cue

**DOI:** 10.3389/fpsyg.2020.00854

**Published:** 2020-05-15

**Authors:** Orit Baruch, Liat Goldfarb

**Affiliations:** ^1^The Institute for Information Processing and Decision Making (IIPDM), University of Haifa, Haifa, Israel; ^2^E. J. Safra Brain Research Center for the Study of Learning Disabilities, University of Haifa, Haifa, Israel

**Keywords:** visual attention, exogenous, reflexive attention, Mexican Hat modulation, visual acuity, bottom-up processes

## Abstract

Classical models of exogenous attention suggest that attentional enhancement at the focus of attention degrades gradually with distance from the attended location. On the other hand, the Attentional Attraction Field (AAF) model (Baruch and Yeshurun, 2014) suggests that the shift of receptive fields toward the attended location, reported by several physiological studies, leads to a decreased density of RFs at the attentional surrounds and hence the model predicts that the modulation of performance by spatial attention may have the shape of a Mexican Hat. Motivated by these theories, this study presents behavioral evidence in support of a Mexican Hat shaped modulation in exogenous spatial tasks that appears only at short latencies. In two experiments participants had to decide the location of a small gap in a target circle that was preceded by a non-informative attention capturing cue. The distance between cue and target and the latency between their onsets were varied. At short SOAs the performance curves were cubic and only at longer SOAs- this trend turned linear. Our results suggest that a rapid Mexican Hat modulation is an inherent property of the mechanism underlying exogenous attention and that a monotonically degrading trend, such as advocated by classical models, develops only at later stages of processing. The involvements of bottom-up processes such as the attraction of RFs to the focus of attention are further discussed.

## Introduction

The term spatial attention refers to selection processes that grant priority to information gathered at the attended location (e.g., Yantis, 1996). Two different types of attentional control have been identified: reflexive (exogenous or stimulus driven) and voluntary (endogenous or goal-driven) (e.g., Jonides, 1981). According to the common view the location to which exogenous attention is directed is selected first by some selection mechanisms that determine the most salient stimulus in the visual field, in a bottom-up analysis of the visual data (e.g., Koch and Ullman, 1985; Wolfe, 1994; Itti and Koch, 2001) whereas voluntary attention is directed according to the goals of the observer (e.g., Jonides, 1981; Maringelli and Umilta, 1998).

Classical views of spatial attention, whether voluntary or reflexive, suggest that attention leads to perceptual facilitation and improved performance, and that this effect is diminished with distance from the focus of attention. Several metaphors were proposed to support this notion, such as the spotlight (e.g., Posner, 1980; Posner et al., 1980), zoom lens (e.g., Eriksen and Hoffman, 1972; Eriksen and St.James, 1986), or gradient (e.g., LaBerge, 1983; Shulman et al., 1985; LaBerge and Brown, 1986; Henderson and Macquistan, 1993). However, recently, some studies of attention found evidence for a suppressive annulus surrounding the enhancement at the focus of attention, both at the physiological level (e.g., Müller and Kleinschmidt, 2004; Hopf et al., 2006; Sundberg et al., 2009) and at the behavioral level (e.g., Eriksen et al., 1993; Cave and Zimmerman, 1997; Caputo and Guerra, 1998; Mounts,2000a, b; Turatto and Galfano, 2001; Cutzu and Tsotsos, 2003). Such evidence led some studies to suggest that the attentional modulation can have a Mexican-Hat profile: i.e., enhanced performance at the focus of attention, accompanied with degraded performance at nearby locations^1^ (e.g., Müller et al., 2005; Hopf et al., 2006; Caparos and Linnell, 2009; Heinemann et al., 2009).

### Possible Explanations to the Mexican-Hat Attentional Modulation

One possible explanation to the Mexican-Hat shaped attentional modulation is that a top-down facilitating mechanism at the selected location is accompanied by a top-down suppressive mechanism of surrounding areas (e.g., Tsotsos et al., 1995; Luck et al., 1997b; Boehler et al., 2011). For example, in a recent study by Boehler et al. (2009) event-related magnetic field (EMRF) was measured to assess the effect of distance and SOA between a target and a following probe. Significant surround attenuation in the EMRF signal was found only for an SOA of 250 ms. This was taken as evidence for a top-down suppression mechanism. However, the task in Boehler et al. (2009) was a search task for a luminance pop-out target. Thus, the top-down allocation of voluntary attention in search for the bright target in this task was probably accompanied by the capture of reflexive attention by the salient target as well as by the onset of the probe. Further, it is not clear how the distractors in the search task affected the perceptual and attentional processes, so it is not completely clear what attentional effects were measured in this study.

An alternative account is that enhancement at the attended location is achieved at the expense of the immediate surrounds due to spatially local tradeoffs in processing capacity (Bahcall and Kowler, 1999; Baruch and Yeshurun, 2014). The Attentional Attraction Field (AAF) model of attention (Baruch and Yeshurun, 2014), proposes that the modulation of performance by spatial attention may have the shape of a Mexican Hat (MH). The model is based on the conception of attention as an attraction field: relying on several physiological studies that found that attention is accompanied by the shift of receptive fields (RFs) toward the attended location (e.g.,Connor et al., 1996, 1997; Womelsdorf et al., 2006, 2008; Quraishi et al., 2007), the AAF model suggests that the allocation of attention to a location attracts (shifts) RFs toward that location. Computational model simulations have shown that a non-linear attraction of RFs to the focus of attention results in a MH shaped density function of RFs, such that with attention, the density of RFs is higher at the focus of attention and lower at the surrounds as compared to their density without attention (Baruch and Yeshurun, 2014). The density of RFs is linked to the density of neurons that are available to process a stimulus and hence may be viewed as an indicator of the available processing resources. Moreover, the MH modulation of available processing resources may further be linked to the MH modulation of performance, and should statistically be reflected as a cubic trend of a task’s performance curve.

### Evidence in Support of the Mexican-Hat Attentional Modulation

Motivated by the idea that one role of attention is to facilitate the selection of task relevant information while ignoring irrelevant distracting information, the above mentioned studies (e.g., Müller et al., 2005; Hopf et al., 2006; Caparos and Linnell, 2009; Heinemann et al., 2009) included distractors along with the target and manipulated voluntary endogenous attention to target attributes or locations in high level perceptual tasks. For example, in a study by Müller et al. (2005) subjects had to discriminate target letters that appeared at a fixed location within an array of distracting letters. Müller et al. (2005) found that distractor letters at a distance of 4.7° from a target letter interfered more with the discrimination of the target letter than at a distance of 2.5°. Clearly, task performance in the above mentioned studies could benefit from an attentional modulation by which enhancement at the target location is accompanied by suppression at the nearby distractor locations. Thus, it remained unclear whether the MH attentional modulation is restricted to cases in which distractors are needed to be inhibited in nearby locations and whether it is restricted to voluntary attention. Recently, measuring MEG response in a Visual Search task, Boehler et al. (2011) found that surround attenuation was not affected by the presence or absence of distractors. However, in this study, voluntary (endogenous) attention to a colored target was manipulated and accordingly, stimuli display times, and target-cue intervals were long (in the order of hundreds of milliseconds).

### The Current Study

The current study was designed to examine how performance is modulated when reflexive, exogenous attention is captured by a non-informative cue. In contrast to previous studies, where the MH modulation was examined when voluntary attention was directed to the location of the target (e.g., Müller et al., 2005; Hopf et al., 2006; Caparos and Linnell, 2009; Heinemann et al., 2009), in the current study, the location of the attention capturing cue does not predict the location of the target, and therefore task related performance cannot benefit from the inhibition of the surrounds of this location.

Some previous studies did examine the attentional modulation in response to attention capturing cues: color singletons (Caputo and Guerra, 1998; Mounts,2000a, b; Turatto and Galfano, 2001) orientation singleton (Mounts,2000a) or abrupt onsets (Mounts,2000b). In these studies, a target was embedded in an array of non-target elements and performance was measured in a discrimination task, as a function of the distance between the target and the attention capturing distractor. It was found that when the distance between the target and distractor decreased, performance decreased, suggesting that the capture of attention induces inhibition at nearby locations. However, the setting of the experiments in these studies involved a multitude of stimuli. Under such conditions it is difficult to isolate local low-level processes and to distinguish between bottom-up and top-down influences. Moreover, all the mentioned studies examined the capture of attention in visual search tasks. Visual search, by definition, involves top-down attention, again, rendering it difficult to isolate the role of top-down versus bottom-up processes in these tasks. In contrast to these studies, the current research examined attentional modulation in a simple visual acuity task that did not involve distracting information at all.

Visual acuity is a perceptual quality that is known to be affected by spatial attention. It was reported that spatial resolution is enhanced at the attended location (e.g., Yeshurun and Carrasco, 1998, 1999; Golla et al., 2004). Moreover, Montagna et al. (2009) found that this enhancement is accompanied by degradation at an unattended location on the other side of the display (however, the shape of the modulation of acuity by exogenous attention was not examined). As mentioned above, a possible explanation to this behavioral phenomenon may be found in the AAF model (Baruch and Yeshurun, 2014). Specifically, the concentration of RFs at the focus of attention, suggested by the model, may lead to enhanced acuity at the focus of attention accompanied by decreased acuity at the surrounds.

In the current study, two experiments examined the modulation of visual acuity when a non-informative attention capturing transient pre-cue summoned reflexive attention. Performance was measured when both distance and SOA (Stimulus Onset Asynchrony) between cue and target were manipulated. The hypotheses that were examined were (a) Modulation of performance, as demonstrated in a visual acuity task, has the shape of a Mexican-Hat. This is an inherent property of reflexive exogenous attention and does not depend on the presence of distractors in the display: (b) The Mexican-Hat profile is initiated by lower processes and hence can be found at short latencies. The classical pattern by which enhancement at the focus of attention degrades gradually with distance might be a result of higher processes that are activated at longer latencies.

## Experiment 1

The purpose of this experiment was to examine hypotheses regarding the way performance is modulated following an attention capturing cue in the context of a visual acuity task. Participants had to perform a gap detection task: a circle with a small gap either at its top or at its bottom was presented for a short duration and participants had to decide what was the position of the gap. Each trial began with the display of 12 placeholder frames for 500 ms, followed by an offset of a randomly selected frame (non-informative exogenous cue). Two factors were manipulated: (a) The distance between the pre-cue and the target, and (b) the Stimulus Onset Asynchrony (SOA) between the onsets of the pre-cue and the target. The presentation of the target followed the onset of the pre-cue (i.e., the offset of the frame) by one of three equally probable SOAs: 133, 167, or 466 ms.

If indeed the Mexican Hat shape of the attentional modulation is an inherent property of the attentional mechanism and is not dependent on the presence of distractors near the target, performance in this experiment should display a Mexican Hat profile. Furthermore, if indeed this property of the attentional mechanism is a consequence of fast processes, such as the local attraction of RFs toward the focus of attention as predicted by the AAF model (Baruch and Yeshurun, 2014), we should expect to see the Mexican Hat shaped modulation at short latencies (short SOAs)^2^.

### Method

#### Participants

Fifteen students from the University of Haifa participated in the experiment in return for course credits or payment ($7). Written informed consent was obtained from all participants. The Ethical Committee of Haifa University approved all the procedures in this study. All participants had normal or corrected to normal vision with no declared learning disabilities or ADHD. The participants were naive as to the purpose of the experiment.

#### Stimuli and Apparatus

The stimuli were presented using MATLAB and the Psychophysics Toolbox extensions (Brainard, 1997). An HP Compaq computer with an Intel core i7-2600 central processor was used to present stimuli and to collect the data. Stimuli were presented on a 22-inch Samsung monitor which participants viewed with their head on a chinrest from a distance of 57 cm. The fixation mark was a 0.3°×0.3° white cross presented at the center of a black background. Twelve gray squares (RGB: 180,180,180) of side 1.2°, served as placeholders and were displayed throughout the experiment. They were placed on an imaginary circle, equidistant from fixation (4.5°), with an interspacing of 30° of arc, starting from 0°. The brief offset of one of the placeholders was used as a pre-cue. The target was a white circle with a small gap either at the circle’s top or at its bottom. The radius of the target was 0.25°, and it appeared for a limited duration centered within one of the placeholders. Across participants, the size of the gap was adjusted to their performance in a practice session that preceded the experiment and was set at a value between 20° and 40° of the target’s arc which is equivalent to 0.087°–0.17° of visual angle.

#### Procedure

**FIGURE 1:**
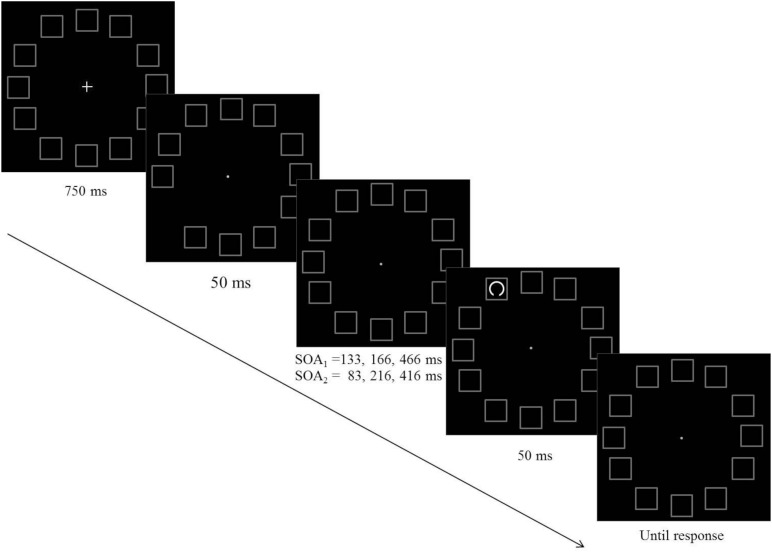
A schematic example of the sequence of events in Experiments 1 (SOA_1_) and Experiment 2 (SOA_2_).

Each trial began with the presentation of the central fixation cross and the 12 placeholders for 750 ms followed by a non-informative pre cue, which was the offset of one of the placeholders for 50°ms^3^. Each of the placeholders had an equal probability to be selected as the pre-cue. The presentation of the target followed the pre-cue by one of three equally probable SOAs: 133, 167, or 466 ms. It could appear in any of the placeholders with an equal probability, thus the spacing between the pre-cue and the target could be 0°, 30°, 60°, 90°, 120°, 150°, or 180° of arc, which is equivalent to 0°, 2.3°, 4.5°, 6.4°, 7.8°, 8.7°, or 9° of visual angle. The gap appeared equally often at the top or at the bottom of the target circle (see Figure 1). The task was to indicate whether the gap was at the top or at the bottom of the target circle and to respond by pressing one of two keys (“1” or “0”, respectively) as quickly and as accurately as possible. The order of the various conditions (SOA, pre-cue location, target location, gap location) was randomized. Each observer participated in 36 practice trials and 864 experimental trials. The size of the gap was set initially to 30° of an arc and was adjusted during the practice session so as to keep the percentage of correct responses in the range of 70–85%. If it exceeded the range, gap size was adjusted by steps of 5°.

### Results and Discussion

To investigate the spatial and temporal distribution of attention, the Inverse Efficiency Score (IES) was calculated. The IES is a combined measure of speed and accuracy, commonly used in attention studies (e.g., Townsend and Ashby, 1978; Akhtar and Enns, 1989; Murphy and Klein, 1998; Kennett et al., 2001; Rossignol et al., 2009; Robertson et al., 2013). Specifically, IES scores were computed as reaction time divided by accuracy, with lower scores reflecting more efficient processing. Trials where RTs were longer than 2 s or shorter from 0.2 s were excluded from the analyses (0.8%).

#### Analysis of the IES Data

A 3(SOA) × 7(pre-cue – target distance) repeated measures ANOVA was performed on the IES measure. The analysis revealed a significant effect of distance (*F*_(6,__84__)_ = 5.68, *p* < 0.001) and a significant interaction between distance and SOA (*F*_(12,__168__)_ = 3. 75, *p* < 0.001).

**FIGURE 2:**
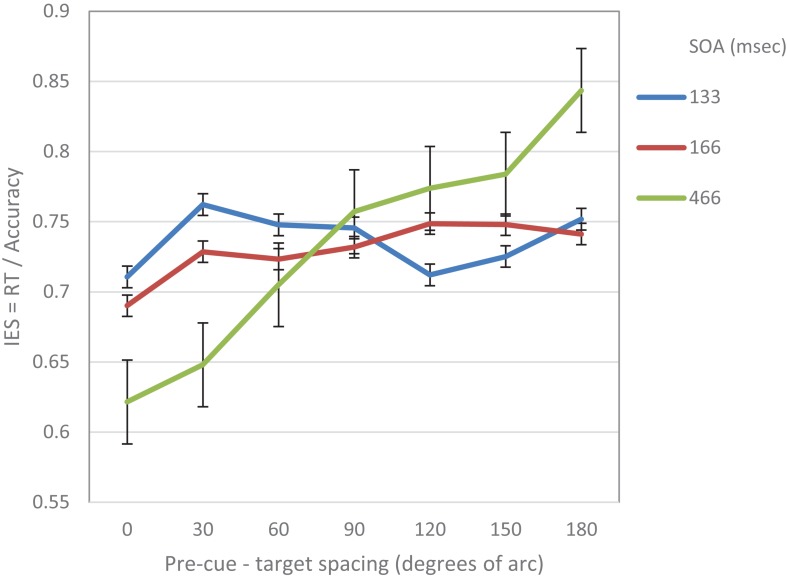
IES (a combined RT-Accuracy measure) in Experiment 1 as a function of pre-cue – target spacing and SOA.

We further performed a trend analysis of the individual curves for cubic and linear trends. At SOA 133 ms a significantly cubic trend was found (*F*_(1,__14__)_ = 7.17, *p* = 0.018). The linear trend was not significant (*F* < 1). At SOA = 166 ms the trend was not significantly cubic (*F* < 1) nor linear (*F*_(1,14)_ = 2.99, *p* = 0.1). At SOA = 466 ms the curve was significantly linear (*F*_(1,__1__–__4__)_ = 12.37, *p* = 0.003) The cubic trend was not significant (*F* < 1) (see Figure 2).

In addition, using curve fitting, we examined for each participant the goodness of fit of the IES curve to each of the two alternative models: the linear or the cubic. IES scores were calculated by dividing the RT at each step by the average accuracy for the SOA and cue-target separation of that step. This calculation was intended to provide a measure that is not based solely on RT, and hence more robust. The result revealed that at the short SOA the fit of the cubic model was significant for 9 of the 15 participants whereas the linear model was significant only for 2. In 6 cases none of the models was significant. These results show that at the short SOA even at the individual subjects level there is support for a cubic performance trend over a linear one.

#### Hemifield Control

As stated above, there is evidence showing that the shift of receptive fields toward the cued location occurs even in the hemifield that is contralateral to the cued location (e.g., Womelsdorf et al., 2006; Klein et al., 2014). Nevertheless, we repeated all the above-mentioned analyses with a hemifield control: only cases in which the cue and the target were in the same hemifield were included in the analysis. The top and bottom locations (at 90° and 270°) were affiliated to both hemifields and thus the analysis included all seven distances (albeit with uneven distribution of trials in the different distances). The analyses yielded qualitatively similar results. Specifically, we found a marginally significant cubic trend (*F*_(1,1__4__)_ = 3.6, *p* = 0.077) at SOA = 133 ms and a significantly linear trend at the long SOA = 466 ms (*F*_(1,1__4__)_ = 13.2, *p* < 0.01). Other trends were not significant, though we did find supporting evidence for suppression at the surrounds of the cued location at SOA = 166 ms: trend analysis of the four nearest locations displayed a significantly quadratic curve (*F*_(1,12)_ = 13.0, *p* < 0.01).

To sum up, the results of this experiment support the prediction that at short SOAs the attentional modulation of acuity displays a cubic trend implying a Mexican hat modulation. This shape seems to be replaced at longer SOAs by a linear trend.

## Experiment 2

In order to examine the change in the shape of the performance curve as a function of SOA and distance between pre-cue and target, Experiment 2 was designed to expand the results obtained in Experiment 1 by using additional SOA values. Specifically, the current experiment examined whether the cubic “Mexican-Hat” like shape of the performance curve will be found at an earlier SOA (less than 100 ms) and to investigate the shape of the curve at intermediate SOA values (between the 166 ms and the 466 ms that were used in Experiment 1).

### Method

The method in Experiment 2 was similar to that of Experiment 1, aside from the following changes: 15 university students participated in this experiment; none of them participated in Experiment 1. The SOAs that were used in this experiment were: 83, 216, and 416 ms. The experiment had 864 trials preceded by 36 practice trials.

### Results and Discussion

#### Analysis of the IES Data

**FIGURE 3:**
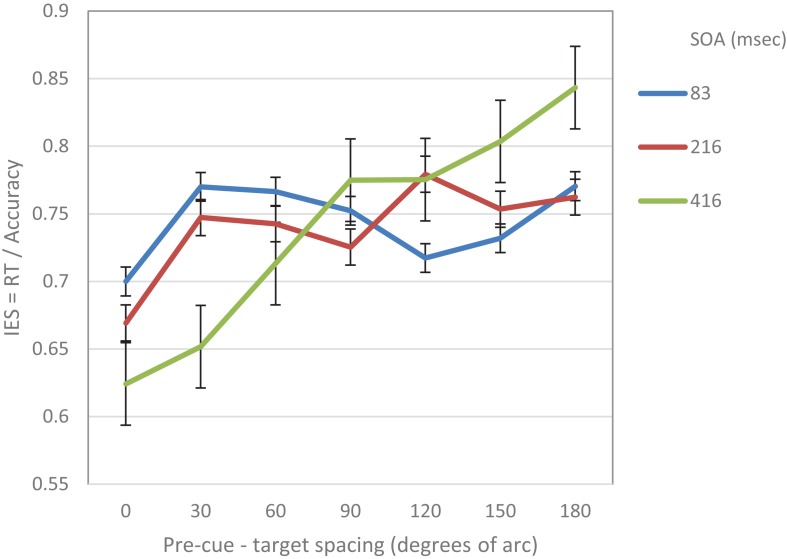
IES curves in Experiment 2 as a function of pre-cue – target spacing and SOA.

As in Experiment 1, the IES scores were calculated for each participant in each condition. Trials where RTs were longer than 2 s or shorter from 0.2 s were excluded from the analyses (0.6%). On this data, repeated measures two-way ANOVA was performed, with SOA and pre-cue – target distance as within subject independent variables. As in Experiment 1, the analysis revealed a significant effect of distance (*F*_(6,84)_ = 5.78, *p* < 0.001) and a significant interaction between SOA and distance (*F*_(12,168)_ = 4.54, *p* < 0.001). There was also a significant effect of SOA (*F*_(2,28)_ = 4.3, *p* < 0.05) (Figure 3). Trend analysis showed that at SOA = 83 ms the IES curve was significantly cubic (*F*_(1,14)_ = 5.9, *p* < 0.05), but not linear (*F* < 1) whereas at SOAs 216 and 416 ms it was significantly linear (*F*_(1,14)_ = 6.57, *p* < 0.05, *F*_(1,14)_ = 14.04, *p* < 0.01; respectively) and not cubic (*F* < 1; *F*_(1,14)_ = 1.7, *p* = 0.22; respectively).

Table 1 summarizes the results of the trend analysis of the IES curves at the various SOAs that were used in both experiments.

**TABLE 1:** A summary of trend analyses of IES curves as a function of SOA, in Experiments 1 and 2.

Trend analyses of performance curvesh		
SOA (ms)/Trend	Linear	cubic
83	*F* < 1	*F*_(1,14)_ = 5.9, *p* < 0.05
133	*F* < 1	*F*_(1,14)_ = 7.17, *p* = 0.018
166	*F*_(1,14)_ = 2.99, *p* = 0.1	*F* < 1
216	*F*_(1,14)_ = 6.57, *p* < 0.05	*F* < 1
416	*F*_(1,14)_ = 14.04, *p* < 0.01	*F* < 1
466	*F*_(1,14)_ = 12.37, *p* = 0.003	*F*_(1,14)_ = 1.7, *p* = 0.22

**FIGURE 4:**
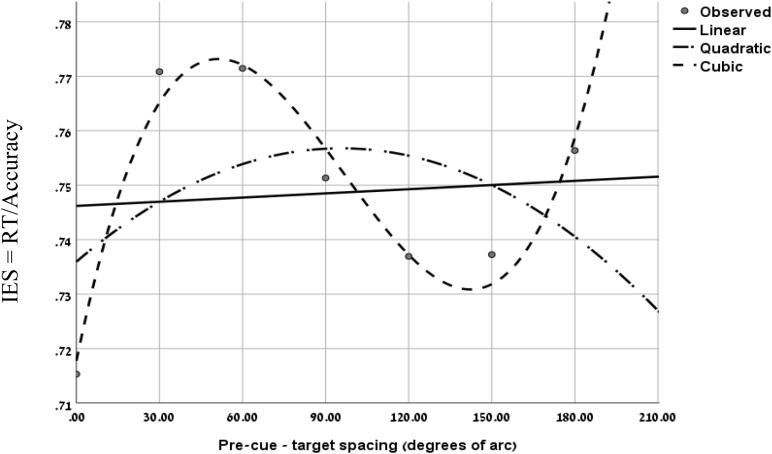
Curve fitting regression on the combined IES data of experiment 1 at SOA = 133 ms and Experiment 2 at SOA = 83 ms, as a function of pre-cue – target spacing.

To further evaluate the validity of the cubic trend at the short SOA in the two experiments, we performed a curve estimation regression for linear, quadratic and cubic models, on the combined IES data of the short SOAs of both experiments, as a function of pre-cue – target spacing (see Figure 4). The analysis of the combined averaged data of all participants revealed that the linear and quadratic curve fitting was not significant (Fs < 1), whereas the cubic regression was significant (*F*_(1,3)_ = 22.93, *p* < 0.05) *with an R2 of.958.* This result further supports the MH hypothesis at short SOAs.

In addition, as in Experiment 1, using curve fitting, we examined for each participant the goodness of fit of the IES curve, to the linear and the cubic models. We found that at the short SOA the cubic trend was significant for 11 participants while the linear trend was significant for 4 out of the 15 participants. In 4 cases none of the models was significant. These results provide further support to the hypothesis of the Mexican-Hat like trend at the short SOAs.

#### Hemifield Control

As in Experiment 1 we repeated the analyses of the IES curves with hemifield control. Again we found qualitatively similar results. At SOA = 83 ms, we found a marginally significant cubic trend (*F*_(1,14)_ = 3.62, *p* = 0.078) whereas at SOA = 216 ms and SOA = 416 ms we found significantly linear trends (*F*_(1,14)_ = 5.63, *p* = 0.03; *F*_(1,14)_ = 10.4, *p* < 0.01; respectively).

To sum up, as in Experiments 1, the results of Experiment 2 support our hypotheses: At short SOAs, the modulation that was exerted by exogenous attention on visual acuity displayed a cubic trend. Interestingly, at longer SOAs the attentional modulation exhibited a different —linear— trend.

### Joined Analyses: Experiments 1 and 2

To further examine the general trends in the two experiments, we joined the data from the two experiments so that three types of SOAs were used: short SAO (83, 133 ms) intermediate SOA (166, 216 ms) and long SOA (416, 466 ms).

On this data we first examined the cubic and linear polynomial contrasts. The analysis of the IES data, revealed a significant cubic trend for the short SOA (*F*_(1,29)_ = 13.45.9, *p* < 0.001), but not for the intermediate and long SOAs *F*s < 1. In addition, the results revealed a significant linear trend for long (*F*_(1,29)_ = 27.36, *p* < 0.001) and intermediate SOAs (*F*_(1,29)_ = 8.6, *p* < 0.01) but not for the short SOA *F* < 1.

**FIGURE 5:**
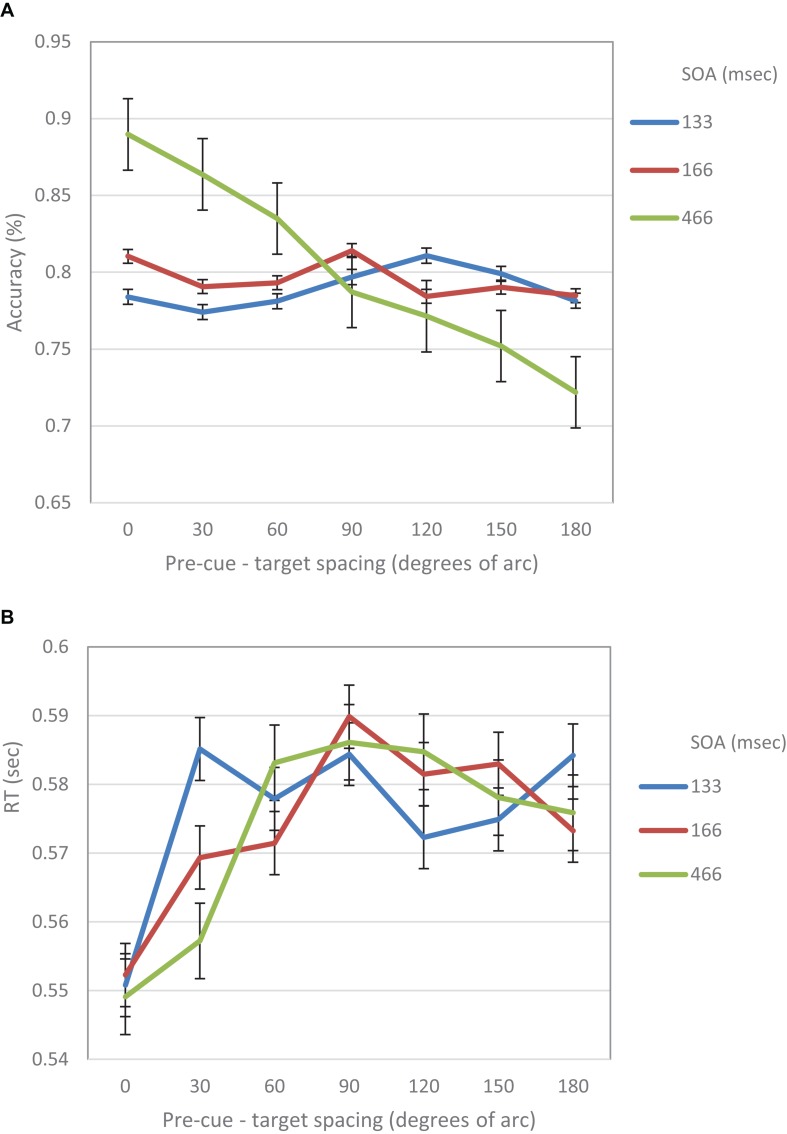
**(A)** Accuracy in Experiment 1 as a function of pre-cue – target spacing and SOA. **(B)** RT in Experiment 1 as a function of pre-cue – target spacing and SOA.

**FIGURE 6:**
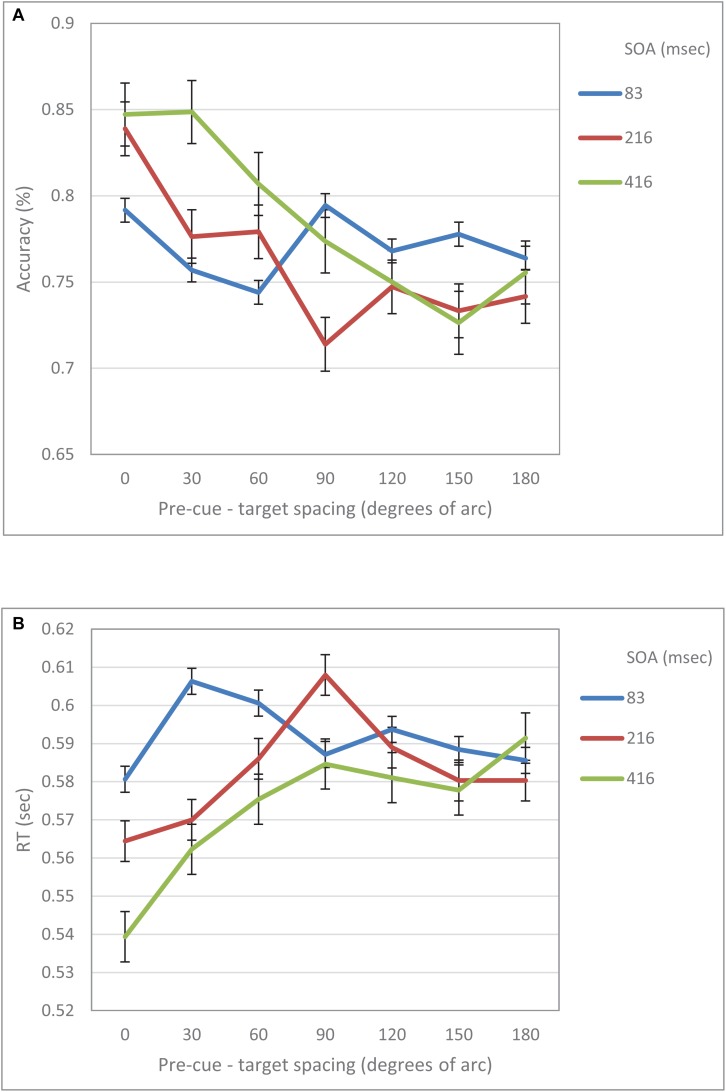
**(A)** Accuracy in Experiment 2 as a function of pre-cue – target spacing and SOA. **(B)** RT in Experiment 2 as a function of pre-cue – target spacing and SOA.

When examined separately, a similar pattern was obtained for both the accuracy and the RT data (see Figures 5, 6). At the short SOA we found a significant cubic trend (*F*_(1,29)_ = 6.53, *p* = 0.016; *F*_(1,29)_ = 11.2, *p* = 0.002, respectively), and a non-significant linear trend (*F*_(1,29)_ < 1; *F*_(1,29)_ = 1.42, *p* = 0.24, respectively), whereas at the long SOA the cubic trend was not significant (*F*_(1,29)_ = 2.87, *p* = 0.1; *F*_(1,29)_ = 1.63, *p* = 0.2, respectively) and the linear trend was (*F*_(1,29)_ = 46.74, *p* < 0.001; *F*_(1,29)_ = 13.3, *p* = 0.001, respectively). Contrasts analyses on data from the same hemifield revealed qualitatively similar results for the IES data as well as for both the accuracy and the RT data. Note, that at the short SOA, these results indicate the same suppression zone for all measures, a suppression zone that disappears at the long SOA.

Moreover, Bayesian analyses were performed on the combined IES data. The results provide a very strong evidence for a cubic trend at the short SOA, BF_10_ = 30.52, and moderate evidences that the cubic trend *does not emerge* in the intermediate and long SOAs, BF_10_ = 0.20, BF_10_ = 0.15, respectively. In addition, the data provides an extreme evidence in support of a linear trend at the long SOA BF_10_ = 164.90 and a moderate evidence for this trend in the intermediate SOA, BF_10_ = 5.52. In contrast the data provides moderate support that the linear trend *does not* emerge in the short SOA BF_10_ = 0.14

These results expose a change in performance between the short and longer SOAs: at short SOAs they exhibit a local reaction to the cue – an enhancement at the cued location compared to its immediate surroundings, whereas at longer SOAs the benefit at the cued location gets more significant when compared to increasingly distant locations. Hence, these results provide additional support to our prediction of a Mexican Hat modulation at short latencies following an exogenous cue that is transformed to a monotonically degrading trend, only at later stages of processing.

## General Discussion

In this study we examined the modulation of performance in a visual acuity task, following a non-informative *exogenous* attention capturing pre-cue. The IES performance curves found in two experiments, exhibit a clear Mexican-Hat shaped modulation at short SOAs (83, 133 ms) and become linear at the longer SOAs (216, 416, 466 ms). At short SOAs there seems to be a suppression of performance at locations that are near to the location of the attention capturing cue, whereas at the long SOAs performance gradually decreases with distance between cue and target exhibiting a linear trend. Note that performance in different perceptual tasks is known to interact differently with various parameters related to exogenous attention. For example, the Inhibition of Return (IOR) effect is typically found sooner in detection tasks than in discrimination tasks (e.g., Lupiáñez et al., 1997; Chica et al., 2006), Since the task in the reported experiments was a discrimination task, IOR is typically seen at SOAs that are longer than the longest SOA that we used in our experiments, hence, IOR was not expected.

The analysis of RT and accuracy data revealed a similar pattern. Specifically, the accuracy performance at short SOAs displayed a cubic trend which became linear at longer SOAs. These results support our claim that there is a Mexican Hat modulation of visual acuity following an exogenous cue.

Top-down attentional modulations were found typically at delays of about 250 ms (e.g., Luck et al., 1997a; Mehta et al., 2000; Chelazzi et al., 2001; Ogawa and Komatsu, 2004). Moreover, the feed-forward sweep of processing is known to take approximately 100-120 ms to reach higher brain areas (e.g., Bullier, 2001; Schmolesky et al., 1998; Lamme and Roelfsema, 2000). Thus, our results suggest that the surround suppression was a consequence of activity that might occur before any recurrent processes could take place. Of course, these results do not rule out the possibility that a Mexican-Hat modulation could be initiated also by top-down processes; however, they suggest that the involvement of top-down processes in this modulation is not mandatory.

**FIGURE 7:**
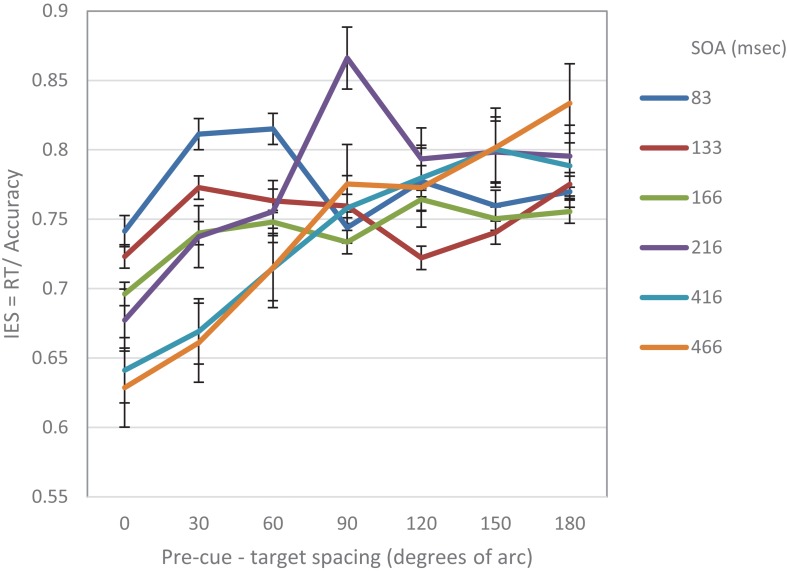
IES curves in Experiment 1 and Experiment 2, as a function of pre-cue – target spacing and SOA.

Figure 7 displays the combined IES curves of both experiments. It seems that there is a gradual effect over time. One clear result is that performance at the cued location is gradually improving and performance at distant locations is gradually degrading. It also seems that the decrease in performance near the cued location gradually disappears. It may be that initially the effect is driven by local sensory interactions, whereas at a later stage, top-down mechanisms are engaged, and replace the local effects.

A possible explanation to the fast Mexican-Hat modulation can be found in the AAF model (Baruch and Yeshurun, 2014). According to the model, the Mexican-Hat modulation may be an outcome of the shift of RFs toward the focus of attention. Such a shift may be initiated by local bottom-up processes that are invoked by local events such as local changes in luminance – as in the case of the pre-cue in the experiments that were performed in this study.

There is evidence indicating that part of the behavioral improvements that follow a transient cue can be explained by local sensory interactions caused by the cue (e.g., Müller and Rabbitt, 1989; Remington et al., 1992; Wright and Richard, 2003; Solomon, 2004). However, results showing that transient cues also degrade performance at un-cued locations compared to neutral locations (Posner, 1978; Posner et al., 1980) were taken as an indicator of some form of suppression exerted by higher level processes. Yet, considering the AAF account, it is possible that the early Mexican-Hat modulation found in this study was mediated by sensory interactions (e.g., Wright and Richard, 2003). Other forms of sensory interactions may also explain the degraded performance at locations proximal to the cue. For example, it is possible that there is some masking of the target by the cue (e.g., Polat et al., 2007) or some form of crowding (e.g., Parkes et al., 2001; Levi, 2008), however, the AAF account provides a parsimonious explanation for both the enhanced performance at the cued location and the degraded performance at its surrounds.

In general, any process takes time to build up. Thus, if indeed the MH modulation observed in our experiments results from the shift of RFs toward the location of the attention capturing stimulus as predicted by the AAF model, we can expect a gradual improvement in performance at the location of the cue, as RFs concentrate at that location (see Figure 7). It is reasonable to assume, that after the “attraction” period, there is a “relaxation” period in which the RFs return to their original location, which can be viewed as their “stable state”. We suggest that the shift of RFs is a local sensory reaction that provides a fast response to the stimulus, and that higher top-down processes are engaged later on. Such a behavior can explain why a Mexican Hat profile would disappear at later SOAs.

The current study has some limitations concerning the control of eye movements. Although in our experiments, participants were instructed to maintain fixation at the fixation point, it is possible that they did moved their eyes. However, since eye movements are known to occur at latencies of 150–250 ms (e.g., Rashbass, 1961), they cannot explain our main result regarding the Mexican-Hat modulation found at short latencies.

In addition, in the current study we didn’t use a dedicated neutral condition in our experiments. A neutral condition is typically used to measure cost/benefit effects as a function of cue validity (e.g., Posner, 1978). In typical cuing paradigms, a valid cue predicts the location of an upcoming target, an invalid cue is distant from the target location (typically at the contralateral visual field) and a neutral cue is not informative of target location, but only serves as a target-onset warning signal. There are several typical methodologies that are used to implement the neutral condition. For example, the cue appears at a location which is in between the Valid and the Invalid cue locations (i.e., at the center of the visual field, e.g., Posner, 1978), all possible target locations are cued simultaneously (a multiple cue condition, e.g., Posner and Cohen, 1984; Wright, 1994), or a background flash is used (cf. Mackeben and Nakayama, 1993). However, there are indications that there might be sensory interactions between the “neutral” cue and the target (e.g., Wright et al., 1995). Therefore, when investigating the assumption that the phenomena related to exogenous attention are based on low-level bottom-up neuronal modulations, we were reluctant to inserting another source of unknown influence.

As was mentioned earlier, in the current study we were interested in examining the hypothesis that a Mexican-Hat modulation can be found at short latencies, in tasks that employ exogenous attention. Indeed, the results of this study provide support to this hypothesis. Finding a MH modulation when reflexive attention is employed may suggest that the MH modulation is an inherent attribute of the mechanisms underlying spatial exogenous attention. Moreover, finding a Mexican-Hat modulation at short latencies may indicate that it is a consequence of bottom-up processes.

In addition, the results show that at longer latencies the shape of the performance curve changes. At longer SOAs we found evidence to a linear trend, with performance gradually degrading with increasing distance from the focus of attention. Note that this pattern was predicted by all classical theories of reflexive attention (e.g., Henderson and Macquistan, 1993; LaBerge, 1983; Shulman et al., 1985; LaBerge and Brown, 1986). However, it seems that this pattern is not immediate and develops relatively slowly, at around 170 ms from the attention capturing cue or even later.

## Data Availability Statement

The datasets generated for this study are available on request to the corresponding author.

## Ethics Statement

This study was approved by the ethics committee of the University of Haifa, Israel, and conformed to its standards. Written informed consent was obtained from all participants.

## Author Contributions

OB and LG contributed to conception and design of the study. OB programmed the experiments. OB and LG performed the statistical analysis. OB wrote the first draft of the manuscript. LG wrote sections of the manuscript. Both authors contributed to the manuscript revision, read and approved the submitted version.

## Conflict of Interest

The authors declare that the research was conducted in the absence of any commercial or financial relationships that could be construed as a potential conflict of interest.

## Footnotes

1 The Mexican-Hat modulation is a term borrowed from wavelet theory. It is in fact the normalized second derivative of a Gaussian function and called so due to its sombrero shape.

2 In our experiment, some of the pre-cues appear at locations that are contralateral to the location of the target. Nevertheless, we expected to find the MH modulation, as some studies found that the attentional shift of RFs occurs even in the hemifield that is contralateral to the attended location (e.g., Womelsdorf et al., 2006; Klein et al., 2014).

3 Note, that the reappearance of the placeholder may also be viewed as an abrupt onset and thus further serve to capture attention toward the same location.
